# Criminalizing genomic discrimination: protecting human dignity under UAE and French law

**DOI:** 10.3389/fmed.2026.1944734

**Published:** 2026-10-07

**Authors:** Fatiha Gourari, Mohammad Amin Alkrisheh, Abouelwafa Ibrahim, Nadia Akawi, Enas Mohammed AlQodsi

**Affiliations:** 1 College of Law, United Arab Emirates University, Al Ain, United Arab Emirates; 2 Department of Genetics and Genomics, College of Medicine and Health Sciences, United Arab Emirates University, Al Ain, United Arab Emirates

**Keywords:** comparative legislation, criminal protection, genetic data, genomic discrimination, genomic research ethics, human dignity, UAE human genome law

## Abstract

**Introduction:**

The rapid advancement of genomics and biotechnology has introduced complex legal and ethical challenges, foremost among them genomic discrimination: the use of individuals' genetic traits, family medical history, or inferred genetic status to make adverse decisions in employment, insurance, access to services, or other legally significant contexts. Such practices undermine human dignity, equality, privacy, and trust in institutions.

**Methods:**

Using descriptive–analytical and comparative methods, we analyze the adequacy and effectiveness of criminal–law protections against genomic discrimination in the United Arab Emirates (UAE) and France. We examine the scope of protected genetic information, the constitutive elements of the offense, applicable sanctions, attempted discrimination, aggravating circumstances, and emerging risks arising from familial and forensic uses of genetic data.

**Results:**

Both jurisdictions prohibit discrimination based on genetic characteristics, but important gaps remain regarding attempted offenses, aggravating circumstances, inferred genetic information, and the expanding use of familial and genealogical genetic data. In the UAE, the Human Genome Law expressly prohibits and criminalizes genomic discrimination, while French law prohibits discrimination based on genetic characteristics through its Civil and Penal Codes.

**Discussion:**

We recommend clearer criminal provisions addressing attempts, proportionate and cumulative sanctions where appropriate, explicit aggravating circumstances, and safeguards against discriminatory uses of inferred, familial, forensic, and genealogical genetic information. These reforms would strengthen protection of human dignity.

## Introduction

The world is witnessing unprecedented developments in genomics and biotechnology, opening vast horizons for the advancement of healthcare services, personalized medicine, and the enrichment of scientific research. These technological breakthroughs have enabled the early detection and prevention of diseases, the identification of genetic predispositions, and the design of targeted therapies, thereby transforming medical practice and public health strategies. At the same time, such rapid progress has generated profound legal, social, and ethical challenges. Among these challenges, genomic discrimination stands out as a particularly pressing issue. Genomic discrimination refers to the practice of making decisions that affect individuals' professional, economic, health, or social lives based on their genetic characteristics, family medical history, or inferred genetic status. Such practices constitute a direct violation of the right to human dignity, as they transform private genetic information—a resource meant to enhance health and scientific understanding—into a tool for exclusion, marginalization, or unjust treatment (1).

The danger of genomic discrimination is compounded by the highly sensitive nature of genomic data, which not only reveals information about an individual's health or physiological traits but may also have implications for family members and descendants. The misuse of such information can result in far-reaching consequences, from limiting access to employment or insurance to creating social stigmatization and perpetuating inequality. In the global context, these risks underscore the ethical imperative to establish robust legal frameworks that both safeguard human rights and encourage responsible scientific research.

Genomic discrimination is not confined to information obtained through a formal genetic test. Genetic status may also be inferred from family medical history or from the known diagnosis of a biological relative. In a condition such as Huntington disease, for example, knowledge that a parent carries a pathogenic variant may reveal a substantial inherited risk for the child even before the child undergoes testing. By contrast, many other genetic findings, including cancer-susceptibility variants such as BRCA1, are probabilistic rather than determinative and must be interpreted in light of penetrance, age, family history, environment, and other clinical factors (2, 3). A legal framework limited to laboratory test results may therefore leave individuals exposed to adverse decisions based on inferred genetic risk.

This broader understanding is reflected in U.S. federal law, where genetic information under the Genetic Information Nondiscrimination Act (GINA) includes family medical history, information about genetic tests of family members, and requests for or receipt of genetic services (4). The distinction is important because an employer, insurer, or other decision-maker may obtain or infer genetic risk without ever requesting a DNA sequence or a formal genetic-test report.

The importance of this study lies in its examination of a contemporary issue at the intersection of human rights, bioethics, and criminal law, an issue that remains relatively underexplored in Arab legal scholarship despite receiving increasing attention within international legal and ethical discourse. By analysing the criminal law framework governing genomic discrimination, this study seeks to contribute to the development of legal scholarship while strengthening public confidence in legislative safeguards protecting genetic privacy and human dignity. It further emphasizes the necessity of achieving an appropriate balance between protecting fundamental rights and promoting scientific research and biomedical innovation. Particular significance is attached to the enactment of Federal Decree-Law No. (49) of 2023 Regulating the Use of the Human Genome in the United Arab Emirates, together with the French legal framework, which expressly criminalizes discrimination based on genetic characteristics. These legislative developments provide an appropriate comparative basis for evaluating the effectiveness of existing criminal protection, identifying legislative gaps, and proposing legal reforms capable of safeguarding human dignity while supporting responsible scientific progress.

Furthermore, genomic discrimination poses not only legal and ethical challenges but also practical obstacles to the advancement of medical and scientific research. The fear of being subjected to discriminatory practices may deter individuals from participating in genetic studies, undergoing genetic testing, or seeking diagnostic genetic services, ultimately hindering efforts to understand, prevent, and treat genetic diseases (5). Consequently, the issue has implications that extend beyond individual rights, affecting public health outcomes, scientific progress, and societal well-being.

International scholarship has also demonstrated that fears of genetic discrimination and loss of privacy can affect willingness to undergo medically indicated testing and to participate in research. Seminal work documented concerns about insurance and employment discrimination well before widespread genomic sequencing (6), while later studies have examined genetic discrimination across jurisdictions and the expanding privacy risks created by re-identification and long-range familial searching (7–9). These developments reinforce the need to treat genomic discrimination as a problem of both individual rights and public confidence in genomic medicine.

The research problem can thus be framed through the following central question: To what extent do UAE and French laws provide adequate and effective criminal protection of human dignity against genomic discrimination? This overarching inquiry gives rise to subsidiary questions concerning the clarity of legislative provisions, the scope of their application, the adequacy of prescribed penalties in proportion to the seriousness of the offense, and the potential for strengthening criminal safeguards through explicit provisions addressing attempts and aggravating circumstances.

Despite an expanding body of scholarship on genetic privacy, data protection, and employment or insurance discrimination, there remains limited comparative criminal-law analysis of genomic discrimination under the UAE Human Genome Law and French law. This study addresses that gap by examining whether the two systems provide coherent criminal protection across the definition of protected data, prohibited conduct, evidentiary thresholds, attempted offences, and sanctions. Its contribution is to bring these elements together within a human-dignity-based framework capable of addressing both formal test results and inferred or relational genomic information.

To address these questions, the study employs a descriptive–analytical method to clarify the foundational concepts of genomic discrimination and genomic data, alongside a comparative method to identify points of convergence and divergence between UAE and French law, particularly regarding the constitutive elements of the crime and the penalties imposed. This approach enables a comprehensive assessment of the effectiveness of the criminal framework and the formulation of practical recommendations to strengthen legal protection, ensuring that human dignity is preserved in the face of emerging biotechnological challenges.

To make this comparison transparent, the analysis applies a functional assessment framework comprising five criteria: the scope of protected genomic information; the forms of prohibited discriminatory conduct; the legal contexts in which protection operates; the constitutive elements and evidentiary implications of the offence; and the adequacy of sanctions and available safeguards. The framework is applied to the relevant statutory provisions, judicial and regulatory materials, and international and clinical-ethical sources. This structured approach makes it possible to identify both formal protections and practical gaps in the criminal-law response.

## The nature of genomic discrimination

The international community has devoted considerable attention to adopting conventions to combat discrimination and racism, to uphold the principles of equality and justice for all human beings (10). Article (1) of the International Convention on the Elimination of All Forms of Racial Discrimination defines racial discrimination as: “distinction, exclusion, restriction or preference based on race, colour, descent, or national or ethnic origin which has the purpose or effect of nullifying or impairing the recognition, enjoyment or exercise, on an equal footing, of human rights and fundamental freedoms in the political, economic, social, cultural or any other field of public life.” The International Convention on the Elimination of All Forms of Racial Discrimination of 1965 was adopted and opened for signature, ratification, and accession by United Nations General Assembly Resolution 2106 A (XX) of 21 December 1965. It entered into force on 4 January 1969 in accordance with Article 19.

Article (7) of the Universal Declaration of Human Rights further affirms the principle of equality by providing: “All are equal before the law and are entitled without any discrimination to equal protection of the law. All are entitled to equal protection against any discrimination in violation of this Declaration and against any incitement to such discrimination.” Article (2) of the Universal Declaration of Human Rights, adopted by the General Assembly in Paris on 10 December 1948 under Resolution 217 A (III), sets out for the first time the fundamental human rights that must be universally protected, describing the Declaration as a common standard of achievement for all peoples and nations.

Similarly, Article (26) of the International Covenant on Civil and Political Rights stipulates: “All persons are equal before the law and are entitled without any discrimination to the equal protection of the law. In this respect, the law shall prohibit any discrimination and guarantee to all persons equal and effective protection against discrimination on any ground such as race, color, sex, language, religion, political or other opinion, national or social origin, property, birth, or other status.” The International Covenant on Civil and Political Rights was adopted and opened for signature, ratification, and accession by United Nations General Assembly Resolution 2200 A (XXI) of 16 December 1966. It entered into force on 23 March 1976 pursuant to Article 49.

By contrast, the UAE legislation did not provide a definition of discrimination in the Law Regulating the Use of the Human Genome. Instead, a definition is outlined in Federal Decree Law No. (34) of 2023 Concerning Combating Discrimination, Hatred and Extremism. Article (1) thereof defines discrimination as: “Any distinction, restriction, exclusion or preference between individuals or groups based on religion, creed, sect, race, color, ethnic origin, gender or sex, taking into account the laws in force in the State.”

The French legislation defines discrimination in Article 225-1 of the Penal Code as any distinction made between natural persons based on their origin, sex, family status, pregnancy, physical appearance, surname, place of residence, state of health, disability, genetic characteristics, morals, sexual orientation or identity, age, political opinions, trade union activities, or their actual or presumed affiliation or non-affiliation with a particular ethnic group, nation, race, or religion. Notably, French legislation also extends the definition to legal persons, setting out specific criteria for such discrimination within the same provision.

In legal scholarship, discrimination has been defined as: “A verbal or non-verbal discourse that promotes discriminatory values, as articulated in the Universal Declaration of Human Rights, as well as in various international covenants, treaties, and conventions, together with regional instruments and domestic legislations of states—all of which denounce, prohibit, and even criminalize all forms of discrimination based on sex, race, religion, belief, or the multiplicity and diversity of other affiliations” (11, 12).

It has also been defined as “a system that confers superiority upon one race or lineage over the rest of humankind” (13).

Another definition describes it as: “Any degrading conduct carried out by persons who perceive themselves as superior to others, for any reason implying distinction, preference, or dominance, to achieve aims and purposes connected with satisfying their own desires at the expense of others, thereby undermining the principle of equality and equal opportunity that governs human beings and safeguards their rights and freedoms” (14).

From these perspectives, discrimination may be understood as any verbal or non-verbal discourse that disseminates discriminatory values, as reflected in the Universal Declaration of Human Rights, various international covenants, charters, and conventions, as well as in the domestic legislations of states—all of which denounce, prohibit, and in many cases criminalize every form of discrimination based on sex, race, religion, belief, or the multiplicity and diversity of other affiliations (15).

Accordingly, genomic discrimination constitutes a violation of fundamental human rights, including the rights to equality, non-discrimination, and human dignity. It may be defined as any distinction made among natural persons—whether individuals or groups—in the form of abuse, preference, restriction, or exclusion, based on their genetic or genomic characteristics. Such discrimination manifests in various domains, including employment, education, health insurance, and other areas of public life.

## Genomic discrimination beyond formal genetic testing

For the purposes of legal protection, genomic discrimination should encompass adverse treatment based not only on an individual's own genetic or genomic test result but also on reliable or probabilistic inferences drawn from family medical history, a relative's diagnosis, participation in genetic counseling, or the fact that the person has undergone a genetic-related medical procedure. Otherwise, regulated actors could circumvent a prohibition on requesting genetic test results simply by asking whether a person has ever had reason to undergo genetic testing or counseling.

This concern is particularly visible in prenatal care. Procedures such as amniocentesis may be undertaken for many reasons and may generate information about a fetus rather than evidence of disease in the pregnant person. Treating the mere fact of such testing as a marker of future health risk may therefore produce irrelevant or overbroad adverse inferences. A sound anti-discrimination rule should focus on the prohibited use of genetic information and genetic inferences, not merely on whether a formal test report exists.

## The concept of genomic data

The term genetic data refers to information derived from biological samples that identifies an individual's inherited or acquired genetic characteristics, including unique details relating to physiology or health (16). Genetic testing, however, can reveal far more than physical traits; it may extend to deeper dimensions such as emotional tendencies and personality features (17). With the rapid advancement of scientific techniques, humans increasingly rely on genetic analysis to uncover the secrets of heredity and to study the structure of the human genome. This progress has opened the door to predicting the genetic risks of certain diseases even before birth, as well as to the early detection of predispositions to serious illnesses through advanced genetic screening (18, 19).

Genomic scientists seek to harness these immense possibilities by examining the fine details of human genetic material, intervening, modifying, or reorganizing it when necessary, and even programming it to meet specific requirements. Such possibilities extend beyond purely medical or therapeutic purposes (20), raising sensitive ethical questions concerning the sanctity of life and the permissibility of intervening in the genetic foundations of human beings in ways that may fundamentally alter their biological composition or psychological traits—all under the pretext of pursuing humanitarian medical goals (21, 22).

Genetic information is widely regarded as private and highly sensitive, since every person's genome is unique and cannot be replicated. Indeed, the genome has often been described as a “book of life” that reveals many aspects of an individual's future. Moreover, genetic data is not limited to the person undergoing genetic testing but also extends to their close and distant biological relatives. Thus, accessing the genetic information of one individual inevitably discloses data about the rest of the family. This interconnectedness underscores the need for robust legal safeguards for genetic privacy—an issue that certain legislations have already begun to address.

The relational character of genomic data also limits the practical promise of anonymity. Genomic records that have been stripped of conventional identifiers may still be susceptible to re-identification when combined with demographic information, genealogical databases, or information about relatives. Research has shown that surnames and identities can sometimes be inferred from genomic information using publicly available resources, while long-range familial searches can identify individuals through distant relatives who contributed data themselves (8, 9). Consequently, consent and confidentiality frameworks should not assume that de-identification alone eliminates privacy and discrimination risks for research participants, patients, or their families.

## First: the universal declaration on the human genome and human rights

The General Conference unanimously adopted the Universal Declaration on the Human Genome and Human Rights during its twenty-ninth session on 11 November 1997. Pursuant to Resolution No. 29 C/17, entitled “Implementation of the Universal Declaration on the Human Genome and Human Rights,” the General Conference outlined methodologies for monitoring its implementation. The United Nations General Assembly subsequently endorsed the Declaration by Resolution No. A/RES/53/152, adopted at its fifty-third session on 9 December 1998. [For the Arabic version of the Declaration, see UNESCO's website: Universal Declaration on the Human Genome and Human Rights – Legal Affairs (http://unesco.org).] Universal Declaration on the Human Genome and Human Rights - Legal Affairs (http://unesco.org). For an international perspective on privacy and data protection, see (16, 23).

The Universal Declaration on the Human Genome and Human Rights was the first international instrument to officially address the relationship between genetic engineering technologies and human rights. It sets out principles that embody the global vision of balancing scientific progress in medicine with the safeguarding of human rights. Symbolically, Article (1) of the Declaration recognizes the human genome as the “heritage of humanity.” Article (2) affirms every individual's right to dignity and to respect for their rights, regardless of genetic characteristics. Article (5) requires prior, free, and informed consent before conducting any genetic examination on a biological sample, while also respecting the individual's choice to know—or not to know—the results and their implications.

In addition, Article (7) emphasizes the protection of the confidentiality of genetic information relating to any identifiable person, whether such data are stored or processed for research or other purposes. Article (9) further provides that no restrictions undermining the principles of consent and confidentiality of personal data may be imposed except by law, for legitimate reasons, and within the limits permitted under international law and international human rights law. Although the Declaration does not have binding force like international treaties, it carries considerable weight, as UNESCO member states collectively agreed upon its drafting. This consensus enhances its authority as a key reference document addressing human rights in the context of genetic technologies (23, 24).

## Second: French legislation

Under Chapter One of the French Personal Data Protection Law No. (493), issued on 20 June 2018 and subsequently amended, French legislation adopted the definition set out in Article (4) of the European General Data Protection Regulation (GDPR) of 2016. This definition identifies personal data as “any information relating to an identified or identifiable natural person (‘data subject’); an identifiable natural person can be identified, directly or indirectly, in particular by reference to an identifier such as a name, an identification number, location data, an online identifier or to one or more factors specific to the physical, physiological, genetic, mental, economic, cultural or social identity of that natural person” (25, 26).

The French National Commission on Informatics and Liberty (CNIL) defines genetic data as “any information relating to an identified or identifiable natural person,” while emphasizing that such data “are not personal data like any other.”

French law requires clear and informed consent from the individuals concerned when processing personal data. Moreover, persons responsible for processing the data or having access to it are bound by professional confidentiality. Breach of this obligation exposes them to the penalties stipulated in Article 226-13 of the French Penal Code (27).

## Third: UAE legislation

Federal Decree Law No 49 of 2023 Regulating the Use of the Human Genome constitutes the cornerstone of the United Arab Emirates' legal framework for genomic data. The law recognizes genomic data as a vital tool for advancing healthcare and personalized medicine, reflecting the legislation's commitment to using such information to improve public health, strengthen disease prevention, and provide tailored treatments. This orientation is demonstrated by the definitions of key concepts in Article (1) of the Decree Law.

The genome is defined as “All the genetic material in a living organism, including genes that contain all the biological data it needs to build and sustain another organism like it and distinct from its type. The human Genetic balance is composed of (46) compact molecules of DNA called Chromosomes, in addition to mitochondrial genes. A gene is defined as “the basic unit for the transmission of Genetic traits from parents to children. It consists of a DNA Sequence that occupies a specific position in the chromosome.”

Article (1) of the said law defines genomic data as “Data related to complete Genetic material, obtained as part of Genomic Screening or Scanning and after analysis of a biological sample.” Genetic data is defined as “Data related to a portion of a person's genetic material, which may include DNA Profiling, which is obtained as part of Genetic Screening, Scanning, or DNA Profiling, and after analyzing the Biological Sample.” The law further specifies the institutions responsible for collecting, preserving, storing, and distributing biological samples—such as blood, tissues, and cells—for future use, including blood banks, cord blood and stem cell banks, and similar facilities.

From the foregoing, it is evident that Article (1) of the UAE Human Genome Law reflects a comprehensive and meticulous approach to genetic and genomic data, demonstrating an advanced awareness of their significance in healthcare and scientific research. This approach goes beyond defining basic concepts, distinguishing between different types of tests and screenings to ensure both accurate understanding and proper application. The differentiation between genetic and genomic examinations highlights the need to address each type of information separately, with due regard for ethics and privacy (28).

In line with its commitment to international standards, UAE legislation incorporates the principles of the Universal Declaration on the Human Genome and Human Rights to safeguard human dignity and prevent discrimination based on genetic characteristics. The law prohibits the use of genomic data as a tool for exclusion or discrimination in employment, insurance, or access to services, affirming the principle of equal treatment irrespective of genetic traits. It further mandates prior, voluntary, and informed consent before conducting any genomic test, ensuring that individuals are fully aware of their rights and the intended use of their data, while prohibiting disclosure or exploitation of such data in discriminatory decisions. In this way, the UAE law demonstrates an advanced understanding of the ethical and social implications of genetic data, seeking to protect human dignity while promoting justice and equality in the face of emerging challenges posed by genetic discrimination.

## The legal framework for the criminalization of genomic discrimination

The XIVth International Congress on Criminal Law, held in October 1989, recommended in Resolution (6/5) the regulation of genetic research, the prohibition of arbitrary use of scientific data, and the banning of genetic discrimination in employment and insurance contracts.

Article (2) of the Universal Declaration on the Human Genome and Human Rights affirms every individual's right to respect for dignity, identity, and rights, irrespective of genetic characteristics.

Similarly, Article (1) of the European Convention on Human Rights and Biomedicine, issued by the Council of Europe on 4 April 1997, prohibits discrimination among individuals based on genetic characteristics. This principle was reaffirmed by Article (11) of the Universal Declaration on Bioethics and Human Rights, adopted by the General Conference of UNESCO in October 2003, which prohibits discrimination against any individual or group on any grounds as a violation of human dignity, human rights, and fundamental freedoms.

Thus, international efforts converged on prohibiting the misuse of genetic and genomic information, particularly to prevent discrimination among individuals and groups based on genetic characteristics. This development has prompted many states to enact national legislation pursuing the same objective, including the UAE and France. The UAE legislation prohibits genomic discrimination under Article 25(2) of Federal Decree Law No. 49 of 2023 Regulating the Use of the Human Genome, and criminalized such conduct in Article 36 of the same law.

Meanwhile, the French legislation, drawing from Article 14 of the European Convention on Human Rights—which explicitly linked genetic testing with the prohibition of discrimination—established a general principle in Article 16-13 of the French Civil Code prohibiting discrimination based on genetic characteristics. This principle was reinforced by Law No. 2002-303 of 4 March 2002 on Patients' Rights and the Quality of the Healthcare System, which confirmed the prohibition of discrimination based on genetic characteristics. French legislation criminalizes this conduct under Article 225-1 of the French Penal Code.

## Elements of the crime of genomic discrimination

The crime of genomic discrimination comprises three essential elements: the preliminary condition, represented by genomic data resulting from genomic testing (as explained earlier); the material element, consisting of the conduct and its criminal outcome; and the moral element, embodied in criminal intent, given that the crime is intentional in nature.

## The material element of the crime of genomic discrimination

This section addresses the nature of the act of genomic discrimination and its forms, followed by the resulting criminal outcome.

1. The Act of Genomic Discrimination:

Genomic discrimination primarily infringes upon the right to privacy, the confidentiality of genomic information, and informed consent concerning such data. It constitutes a misuse of genomic data, which may negatively affect an individual's rights to healthcare, employment, education, or other entitlements.

Genomic discrimination may occur when genomic information is used beyond the lawful purposes for which it was collected, resulting in unequal treatment of individuals based on their genetic or genomic characteristics, such as the risk of genetic disorders. In essence, genomic discrimination involves differentiation among individuals according to their genetic traits. This differentiation may take various forms depending on the right infringed, most notably discrimination in employment, insurance, education, and similar areas.

Accordingly, the criminal conduct constituting genomic discrimination consists of using genomic or genetic data in a manner that results in differential treatment of individuals based on hereditary characteristics, racial origins, or lineage, which restricts rights granted under applicable laws, such as access to insurance, employment, promotion, or education. The criminal outcome is the realization of discrimination in these areas as a direct consequence of the use of genomic data derived from genomic testing.

It is noteworthy that the UAE legislation, under Article 19(1)(a) of the Human Genome Law, prohibits employers and employing entities from subjecting job applicants to genomic or genetic testing aimed at discovering or predicting their susceptibility to diseases for the purpose of making employment decisions based on such results.

By contrast, Article 19(2) permits employers or employing entities to conduct voluntary genomic or genetic testing of job applicants or current employees, provided that the individual consents and that a scientifically established causal relationship exists between the person's genetic predisposition and occupational diseases, accidents, or injuries likely to result from performing the specific job.

This exception is designed to safeguard individuals' health by protecting them from potential diseases or injuries linked to their genetic traits in the course of their professional duties.

In this context, the U.S. federal Genetic Information Nondiscrimination Act (GINA) of 2008 likewise prohibits the use of genetic information in employment (Articles 202–213) and health insurance (Articles 101–106). The Act allows employers to conduct genetic testing of employees voluntarily to assess the impact of workplace conditions on health, but does not extend this provision to job applicants (29).

GINA establishes civil, rather than criminal, protections. In employment, Title II is enforced through the Equal Employment Opportunity Commission and incorporates remedies modeled on federal employment-discrimination law, including equitable relief and, where legally available, compensatory and punitive damages subject to statutory limits. It is therefore inaccurate to characterize GINA as creating a schedule of criminal fines for genetic discrimination. Its significance for the present comparative analysis lies in the breadth of the protected concept of genetic information—including family medical history—and in the fact that federal protection remains limited to employment and health insurance rather than all insurance markets (4, 29, 30).

By contrast, the UAE Human Genome Law, under Article 20, prohibits insurance institutions from subjecting applicants for insurance coverage to genomic or genetic testing for the purpose of detecting or predicting disease susceptibility, or from making such testing a condition for granting insurance services. It also forbids requesting or using the results of any prior genomic or genetic tests of insurance applicants.

UAE legislation criminalizes the violations set out in Articles 19 and 20 relating to employers and insurance institutions under Article 35 of the same law, prescribing penalties of imprisonment ranging from one month to three years and fines between AED 50,000 and AED 1,000,000, or either of these penalties.

In light of the foregoing, the misuse of genomic or genetic data that results in differentiation among individuals and infringes upon their rights may stem from previously obtained genetic information. In such cases, the crime of genomic discrimination materializes once actual differentiation between individuals occurs. Alternatively, it may arise from information obtained through genetic or genomic testing that employers or insurance institutions unlawfully require from job applicants or insurance seekers, where the results are misused for discriminatory purposes—such as determining employment eligibility, denying insurance coverage, or imposing excessive premiums. In these circumstances, two distinct violations are involved: the unlawful imposition of testing and genomic discrimination. However, given their inseparable nature, they are treated as a single crime, with the severest penalty applied to the offender in accordance with Article 89 of the UAE Penal Code.

## Criminal outcome

The criminal outcome of the offense of genomic discrimination lies in the misuse of genetic or genomic data in a manner that results in differentiation among individuals based on their hereditary traits, genetic characteristics, racial origins, or lineage, leading to the restriction or deprivation of rights legally guaranteed to them.

The UAE legislation has adopted a broad approach regarding the individual rights affected by the crime of genomic or genetic discrimination, without limiting them to specific rights such as employment or access to insurance coverage, as U.S. federal law does under GINA. This broader formulation is more balanced and appropriate, as it ensures wider legal protection for individual rights against genomic discrimination.

This approach also aligns with the principle of “prohibition of discrimination based on genetic characteristics” as recognized by the French legislation in Article 225-1 of the Penal Code, which includes genetic characteristics among the prohibited grounds of discrimination, and in the 2002 Patients' Rights and Quality of the Health System Act, which amended the Civil Code by adding Article 16-13 to affirm non-discrimination against persons with genetic characteristics.

The primary areas in which individual rights are most affected by genomic discrimination include employment, health insurance, and education (31).

The legal problem is not confined to health insurance. In the United States, GINA protects against discrimination in health insurance and employment, but does not extend the same federal protection to life, disability, or long-term-care insurance; state laws may provide additional safeguards (30). This regulatory gap is important because predictive genetic information may influence access to economically significant products even where no current disease is manifested. Comparable concerns may arise in business-related, property-linked, or other risk-based insurance and contracting contexts, although the relevance of genetic information and the applicable legal limits vary substantially by product and jurisdiction. Canada provides a useful broader comparator: the Genetic Non-Discrimination Act 2017 prohibits requiring a genetic test, or disclosure of a genetic-test result, as a condition of providing goods or services, entering into or continuing a contract, or offering particular contractual terms, and it attaches criminal penalties to contraventions (32). The Canadian model therefore demonstrates that genetic discrimination can be regulated beyond the narrow categories of health insurance and employment.

1. Insurance and Economic Contexts:

Genetic or genomic discrimination in the insurance sector arises when genetic information obtained from genetic or genomic tests is used to deny insurance coverage (33), terminate existing coverage, or impose inflated premiums beyond standard limits based on genetic considerations.

A notable case concerns a young Australian man, James, in his early twenties, who had his life insurance denied by an insurance company after disclosing that he had undergone genetic testing and discussed the results with a genetic specialist. A subsequent test revealed a mutation in the MSH6 gene, which led two other insurance companies to deny him cancer coverage.

Unconvinced by the insurers' risk assessments, James chose to undergo regular colonoscopy screenings, believing this would mitigate his risk of developing cancer. He later filed a complaint with the Australian Human Rights Commission, following which another insurance company eventually granted him full and unrestricted coverage (7, 34).

Similar cases of genetic or genomic discrimination have been documented in several jurisdictions, illustrating the global nature of the issue. In the United States, one of the earliest reported incidents involved Nancy Wexler, a geneticist carrying the gene for Huntington's disease. Before the enactment of the Genetic Information Nondiscrimination Act (GINA) in 2008, some of her family members faced employment and insurance difficulties after their genetic predispositions became known (6). These cases were pivotal in prompting legislative reform to prevent misuse of genetic data in employment and insurance sectors.

In Canada, a landmark series of incidents before the adoption of the Genetic Non-Discrimination Act (2017) revealed that several individuals who voluntarily underwent predictive genetic testing for BRCA1/2 breast cancer mutations were denied life or disability insurance coverage. The resulting public backlash led to the enactment of the 2017 law, which criminalized requiring or using genetic test results as a condition for service or contract (35).

In the United Kingdom, a case reported by the Human Genetics Commission (2011) concerned an employee asked by his employer to disclose whether he had undertaken genetic testing for neurological disorders, raising serious concerns about workplace discrimination and genetic privacy (36). The absence of a specific criminal offense of genetic discrimination does not mean that the United Kingdom lacks safeguards in the insurance context. Since 2018, the UK Government and the Association of British Insurers (ABI) have operated the Code on Genetic Testing and Insurance, which replaced the earlier Concordat and Moratorium on Genetics and Insurance. Under the Code, participating insurers must not require or pressure applicants to undergo predictive or diagnostic genetic testing and, subject to a narrow exception, must not request or take predictive genetic-test results into account. As of the 2025 triennial review and the March 2026 update, the only predictive result that may be requested is a Huntington's disease test for life-insurance applications exceeding £500,000; research-derived predictive genetic-test results are not to be requested or used. The UK model therefore illustrates a significant non-statutory governance mechanism that complements general legislation, while remaining distinct from an explicit criminal-law prohibition of genomic discrimination (37).

In France, a 2019 case before the Conseil d'État involved the denial of insurance to a woman carrying a genetic mutation associated with breast cancer. The court reaffirmed the constitutional principle of equality and the prohibition of discrimination based on genetic characteristics under Article 225-1 of the French Penal Code. This decision strengthened judicial recognition of genetic equality as an integral aspect of human dignity (38).

Collectively, these cases demonstrate how genomic discrimination manifests across jurisdictions and contexts—particularly in insurance and employment sectors—underscoring the need for explicit criminal provisions that safeguard individuals' genetic privacy and uphold the principle of human dignity.

The recurrence of such cases across diverse legal systems reveals a persistent gap between scientific progress and legal protection. Although genomics has advanced rapidly, enabling early detection and prevention of genetic diseases, the legal frameworks in most jurisdictions remain reactive rather than preventive. This imbalance allows private entities—such as insurers and employers—to exploit genetic information for risk assessment or economic gain, thereby transforming a tool of health advancement into an instrument of exclusion.

From a criminal law perspective, the absence of explicit penal provisions addressing genomic discrimination creates a normative vacuum where the violation of human dignity is recognized ethically but not fully sanctioned legally. This vacuum weakens deterrence and undermines public trust in genetic research and health institutions. The ethical promise of genomics—to improve lives through knowledge—becomes overshadowed by fear of misuse, which in turn discourages individuals from undergoing genetic testing, even when medically advisable.

Comparatively, jurisdictions like the UAE, which emphasize the sanctity of human dignity as a constitutional and religious value, are well-positioned to adopt a more proactive stance. Explicit criminalization of genomic discrimination would align with both the constitutional protection of personal rights and the moral foundations of Emirati law, which prioritize equality, justice, and the preservation of human honor. Similarly, the French approach—anchored in the universal principle of égalité and the prohibition of discrimination based on genetic characteristics—demonstrates how the criminal law can serve not merely as a punitive tool but as a normative declaration reinforcing societal values.

Therefore, the convergence of ethical imperatives, human rights principles, and technological realities necessitates a comprehensive criminal framework—one that clearly defines genomic discrimination, criminalizes both direct and indirect forms of it, and incorporates aggravating circumstances when committed by public officials or entities entrusted with sensitive data. Only through such clarity and firmness can the law effectively restore balance between scientific innovation and the inviolable dignity of the human person.

(1) Genomic Discrimination in Employment:

Genetic discrimination in the field of employment manifests in several forms, including refusal to hire an individual based on their genetic information, unjustified dismissal, reduction of salary, restriction of access to training or professional development opportunities, denial of promotion, or involuntary transfer from one workplace to another.

The U.S. Equal Employment Opportunity Commission (EEOC) reported that the number of genetic discrimination cases in the workplace in 2019 reached 209, representing a 5% decrease compared to 2018. These figures do not include cases registered at the state level within the United States.

To reinforce legal protection against genetic discrimination in employment, the French legislation includes Article L.11-45 of the Labor Code, which prohibits the use of genetic characteristics to exclude an individual from recruitment procedures, training, or internships. Furthermore, no employee may be punished, dismissed, or subjected to direct or indirect discriminatory treatment, particularly regarding remuneration, training, reassignment, hiring, qualification, promotion, transfer, or contract renewal, based on their origin or genetic characteristics. In addition, Article 225, paragraph 4 of the French Penal Code criminalizes the refusal to employ or the punishment or dismissal of any person based on their genetic characteristics. Paragraph 5 of the same article criminalizes restricting access to job opportunities, training programs, or the duration of training within an institution when such restrictions are based on any of the prohibited grounds listed in Article 225-1, including genetic characteristics.

## Governmental, forensic, and consumer-generated genetic data

The risks of genomic discrimination also extend beyond employment and insurance to governmental and forensic uses of genetic information. Criminal justice databases such as the U.S. Combined DNA Index System (CODIS) were developed for identity matching using forensic DNA profiles, but familial searching raises a distinct concern because a kinship-based lead can direct investigative attention toward people who are not themselves database subjects. The FBI does not conduct familial searching at the National DNA Index System (NDIS). State practice varies: California and New York permit familial searching under specified procedures, whereas Maryland and the District of Columbia prohibit database familial searching by law (39, 40). This variation illustrates why familial searching should be treated as a separate legal question from an ordinary CODIS match and subjected to explicit safeguards.

Familial searching can generate legitimate investigative leads, but it also creates risks of associative stigma and unequal exposure. A person may become the focus of police inquiry not because of his or her own conduct or individualized evidence, but because a relative's DNA profile is present in a criminal database. This creates a form of genetic suspicion by association—the risk of being 'stained by association' through biological kinship. The concern therefore extends beyond informational privacy to core criminal-justice and human-rights guarantees, including the presumption of innocence and protection against arbitrary or disproportionate search. Repeated interrogation of an individual's genetic relationships merely because a distant relative becomes implicated in an investigation can weaken the requirement that investigative attention be justified by individualized grounds. Where socio-economically disadvantaged or historically overrepresented communities constitute a disproportionate share of forensic DNA databases, kinship-based searching may also extend unequal surveillance to relatives who have never been suspected or convicted of an offense. These effects do not make every familial search unlawful, but they require strict necessity, proportionality, purpose limitation, non-discrimination, auditing, and judicial or independent oversight to prevent inherited or associative suspicion.

Investigative genetic genealogy creates an even wider relational reach. Law-enforcement agencies may generate a dense SNP profile from crime-scene DNA and compare it with consumer or genealogy databases, including services such as GEDmatch and other public or commercial genealogy platforms, to locate distant relatives and construct family trees. The U.S. Department of Justice has issued an interim policy governing forensic genetic genealogical DNA analysis and searching, reflecting the need to balance investigative value with privacy and civil liberties (41). Empirical research shows that long-range searches may identify a target through third cousins or more distant relatives, meaning that one consumer's upload can expose genetic relationships involving many people who never tested or consented to law-enforcement use (9, 42).

Commercial platforms also differ in their policies. Ancestry states that it does not voluntarily permit law-enforcement use of its services and requires valid legal process for disclosure of DNA data, while 23andMe's terms prohibit investigative forensic genealogy uses (43, 44). These contractual protections are important but do not resolve the broader legal question: genetic privacy is relational, and the consent of one database participant may indirectly expose biological relatives. Legislation should therefore distinguish ordinary forensic matching, familial searching, and investigative genetic genealogy, and should regulate each according to the sensitivity, breadth, and intrusiveness of the search.

The UAE provides an important national comparator. Federal Decree-Law No. 39 of 2023 Concerning the Regulation of the Federal DNA Fingerprinting Database specifies the authorized purposes of the federal DNA database, including criminal investigation and identification of perpetrators, victims, missing persons, and unidentified remains, and it identifies the categories and sources from which biological samples may be collected. The legislation also criminalizes use of the database or collected biological samples for purposes outside those legally authorized. In Dubai, the Dubai Police Genome Centre, launched in 2023, includes a Human Genome Section for genetic identification and molecular autopsy and Bioinformatics and Biotechnology Sections that use artificial intelligence for statistical analysis of genomic variants. These developments demonstrate the increasing integration of genomics and AI in forensic practice. However, publicly available official materials reviewed for this study do not establish a specific legal framework authorizing investigative genetic genealogy through consumer genealogy databases. This distinction reinforces the need for transparent rules governing the scope, legal basis, proportionality, oversight, retention, and secondary use of forensic genomic analysis as technological capabilities expand (45, 46).

## Discarded DNA, warrants, retention, and deletion

A related unresolved problem concerns DNA that individuals leave behind on objects such as drinking vessels, food packaging, cigarettes, tissues, or other discarded items. The physical abandonment of an object does not necessarily answer whether comprehensive genomic analysis of biological material found on that object should be treated as a separate and highly intrusive search. Because whole-genome or dense SNP analysis may reveal health predispositions, ancestry, and family relationships far beyond identity, legal systems should distinguish collection of an object from extraction and secondary analysis of genomic information. Maryland's forensic genetic genealogy statute illustrates one regulatory response by requiring judicial authorization for specified forensic genetic genealogical DNA analyses and searches, limiting the databases that may be used, addressing consent for third-party samples, and providing rules on sample destruction and related safeguards (47). These questions are especially important when the person whose DNA is analyzed is later excluded as a suspect or was never charged.

At minimum, proportionality requires clear rules on purpose limitation, retention periods, secondary use, access logs, and deletion or expungement. A profile generated for one investigation should not automatically become a permanent resource for unrelated future searches, particularly where the person is innocent or was never charged. These safeguards are central to preventing forensic genomics from becoming a mechanism of inherited suspicion or discrimination by association (48).

## Legal ruling on attempted intentional genomic discrimination

As the crime of genomic discrimination is classified as an intentional, result-oriented offense, its occurrence requires conduct involving the misuse of genetic or genomic data in a manner that deprives the victim of one or more rights or imposes restrictions upon them. Accordingly, an attempt at this crime may arise where the prohibited conduct has been initiated, but the result does not materialize due to circumstances beyond the perpetrator's control (49). For instance, if an employee sends an email instructing a subordinate to deny an individual insurance coverage or to impose excessive premiums based on genetic information, and the subordinate begins reviewing the case but, due to workload, fails to carry out the instruction, the intended discriminatory result does not occur.

Under the UAE Human Genome Law, genomic discrimination is criminalized as a misdemeanor (Article 36). Pursuant to Article 37 of the UAE Penal Code, punishing attempts requires an explicit legal provision. As the Human Genome Law contains no specific article penalizing attempted genomic discrimination, liability is confined to the completed offense.

The same situation exists under French law, where genomic discrimination is criminalized as a misdemeanor and only in its consummated form, with no express provision in the Penal Code extending criminal liability to attempts.

Given the importance of safeguarding human dignity and the clear possibility of attempting such offenses, it is necessary to criminalize genomic discrimination in its completed and attempted forms alike.

## Challenges in the clinical interpretation of genomic variants

The increasing accessibility of genomic sequencing has substantially improved the identification of genetic variants associated with both rare and common diseases. However, the presence of a pathogenic or likely pathogenic variant in an individual's genome should not be interpreted as a deterministic predictor of disease development. Rather, the clinical manifestation of most genetic conditions depends on a complex interplay between genetic factors, including penetrance, variable expressivity, modifier genes, and polygenic background, as well as non-genetic influences such as environmental exposures, lifestyle, epigenetic regulation, and stochastic biological processes (2, 3). Consequently, many individuals harboring disease-associated variants remain asymptomatic throughout their lifetime, whereas others may develop disease at different ages or with varying degrees of severity.

Recent advances in long-read sequencing and next-generation CRISPR editors further increase the volume, granularity, and clinical relevance of genomic information generated in precision-medicine settings. As integrated pipelines make genetic findings increasingly actionable, the risk that probabilistic or preliminary genomic information may be used outside a legitimate clinical context also increases, reinforcing the need for effective safeguards against genomic discrimination (50).

This complexity is particularly relevant for common multifactorial disorders, such as cardiovascular disease, diabetes, and many cancers, in which genetic variants typically confer susceptibility rather than certainty of disease. Even for highly penetrant variants, individual risk is influenced by additional genetic and environmental modifiers. Therefore, genomic findings should always be interpreted within the broader clinical context, including personal medical history, family history, physical examination, and other diagnostic investigations, rather than in isolation (51).

Moreover, despite rapid advances in genomic technologies, the functional and clinical significance of a considerable proportion of human genetic variation remains incompletely understood. Many variants are currently classified as variants of uncertain significance (VUS), while the pathogenicity of other variants may be reclassified over time as new functional, population, and clinical evidence becomes available (51). Technical limitations of sequencing platforms, bioinformatic pipelines, and variant interpretation can also contribute to false-positive findings or inaccurate classification, emphasizing the need for rigorous validation and adherence to standardized interpretation guidelines.

The interpretation of incidental or secondary genomic findings presents additional clinical and ethical challenges. Although the reporting of medically actionable secondary findings has been recommended under specific circumstances, these findings should not be considered diagnostic in the absence of corroborating clinical evidence. Their interpretation should incorporate family history, segregation analysis, phenotypic assessment, and established evidence-based recommendations to minimize misinterpretation and unnecessary medical interventions (3, 52). Overinterpretation of incidental findings without sufficient supporting evidence may lead to psychological distress, unnecessary diagnostic procedures, and inappropriate clinical decision-making.

Collectively, these considerations underscore that genomic information represents one component of precision medicine rather than a definitive measure of an individual's current or future health. Current scientific evidence does not support the use of genomic data alone to predict disease with certainty or to determine an individual's fitness, employability, insurability, or other personal capabilities. Such applications are scientifically unjustified because genomic risk is inherently probabilistic and continually refined as knowledge of human genetic variation expands. Accordingly, responsible implementation of genomic medicine requires careful integration of molecular findings with clinical evaluation, family history, environmental factors, and evidence-based genetic counseling to ensure accurate interpretation and to prevent discrimination based solely on genetic information (2, 51).

## The mental element in the crime of genomic discrimination

The mental element (mens rea) in the crime of genomic discrimination constitutes a cornerstone for determining criminal responsibility. Although neither UAE nor French legislation explicitly defines the mental element in the relevant provisions, the structure and language of the laws indicate that this offense is purely intentional, excluding the possibility of negligence or recklessness. The act of discrimination cannot arise accidentally; it necessarily presupposes a deliberate human choice and a conscious violation of equality.

From a doctrinal standpoint, the offender's awareness and will must converge toward a single objective: to differentiate, exclude, or restrict another person's rights based on their genetic or hereditary characteristics. Thus, two cognitive dimensions are essential:

Knowledge (science) — the perpetrator must know that the data used are genetic or genomic in nature and relate to the hereditary characteristics of the victim.

Will (volonté) — the perpetrator must intend to use such information to discriminate, marginalize, or deny access to a right or benefit.

If one of these dimensions is absent—such as when the offender uses genetic information without awareness of its nature, or for purposes unrelated to discrimination—the dolus specialis (specific intent) is lacking. Consequently, criminal liability cannot be established.

This interpretation aligns with the general principles of criminal law in both jurisdictions, which require intentionality in crimes involving moral wrongfulness. In the UAE Penal Code, Article 39 defines intent as the “will to commit the act constituting the crime with knowledge of its unlawfulness.” Similarly, Article 121-3 of the French Penal Code distinguishes between intentional and unintentional offenses, reserving criminal sanctions for acts committed “with intent to cause the prohibited result.” Hence, applying these principles to genomic discrimination means that the applicable criminal-law framework implicitly requires proof of intent to misuse genetic data with discriminatory purpose.

Furthermore, the mental element in such crimes reflects a complex form of discriminatory consciousness—not limited to explicit animosity or hatred, but encompassing subtler forms of exclusion arising from institutional or economic motives. For instance, an employer who intentionally excludes candidates based on genetic predisposition to illness acts with discriminatory intent, even if motivated by cost-efficiency rather than prejudice. The law thus focuses not on the motive's morality, but on the intentional choice to treat individuals unequally on a genetic basis.

In comparative perspective, both legal systems would benefit from codifying an explicit definition of intent in the context of genomic discrimination. This would ensure clarity in evidentiary requirements, prevent interpretative ambiguity, and reinforce the principle of legality (nullum crimen sine lege certa). Such clarification would also protect against over-criminalization by distinguishing between legitimate use of genomic data (for research or medical treatment) and malicious or discriminatory misuse.

Ultimately, the mental element in the crime of genomic discrimination encapsulates not only the offender's intent but also the ethical consciousness of the act—a deliberate affront to human dignity through genetic differentiation. Recognizing this dual dimension—legal and moral—strengthens the doctrinal foundation for criminal protection of equality in the genomic era.

## Penalty prescribed for the crime of genomic discrimination

If the crime fulfills all its constitutive elements, it becomes punishable under the law.

1. The UAE legislation prescribes for the crime of genomic discrimination a penalty of imprisonment for a period of not less than one month and not exceeding three years, and a fine of not less than AED 100,000 and not more than AED 500,000, or either of these two penalties (Article 36 of the Human Genome Law).

In addition, Article 42 of the same law provides for complementary penalties, which include:

1. “Confiscation of the tools of the crime, funds, and proceeds arising therefrom. Where it is not possible to rule on confiscation because it relates to the rights of bona fide third parties, the Court shall decide a fine equivalent to the value thereof at the time of committing the crime;
2. Deportation of the foreigner who is convicted of one of the crimes stipulated herein; and
3. Closure of the place in which the offense is committed.”

On the other hand, under French law, the crime of genetic discrimination is punishable by three years' imprisonment and a fine of EUR 45,000 (Article 225-2 of the French Penal Code).

An aggravating circumstance applies if the crime is committed in a place open to the public and access is restricted, in which case the penalty increases to five years' imprisonment and a fine of EUR 45,000.

## Conclusion

Our comparative analysis demonstrates that genomic discrimination directly threatens human dignity, equality, privacy, and meaningful access to employment, insurance, services, and other opportunities. UAE and French law both recognize genetic characteristics as a legally protected basis and provide mechanisms for sanctioning discriminatory conduct. The principal weakness is therefore not the complete absence of legal protection, but the incompleteness and fragmentation of the criminal-law response when discrimination is attempted, inferred from relatives or family history, or generated through emerging forensic, genealogical, and data-intensive uses of genomic information.

We further conclude that genomic discrimination cannot be reduced to misuse of formal medical genetic-test results. Adverse treatment may be based on family medical history, a relative's diagnosis, inferred genetic status, participation in genetic services, or genetic information exposed through consumer, familial, forensic, or genealogical databases. These contexts transform genomic discrimination from a bilateral employment-or-insurance problem into a broader issue of relational privacy, equality, proportionality, presumption of innocence, and protection against arbitrary or discriminatory state and private action.

We identify several concrete weaknesses requiring reform. Neither system expressly addresses every form of attempted genomic discrimination in a manner that fully resolves the risks identified in this study. UAE law permits alternative sanctions for the Article 36 offense—a minimum imprisonment term of one month, a fine, or either penalty—raising questions about whether monetary punishment alone will always provide adequate deterrence for serious or institutionally organized discrimination. The UAE framework also lacks tailored aggravating circumstances for situations such as abuse by a public official or discriminatory conduct directed against a particularly vulnerable person. French law provides a more developed general framework for discrimination based on genetic characteristics, but emerging uses of inferred, familial, forensic, and genealogical data likewise require continued scrutiny.

We recommend that UAE and French law expressly address attempted genomic discrimination where existing general rules do not provide sufficiently clear coverage; ensure that sanctions are proportionate to the seriousness, scale, and institutional context of the conduct; and recognize aggravating circumstances where genomic discrimination is committed through abuse of public authority, systematic misuse of sensitive data, or against vulnerable persons. We also recommend safeguards that extend beyond formal test results to reliable genetic inferences, family medical history, and relational genomic information, together with clear rules for forensic and genealogical uses of DNA concerning necessity, proportionality, judicial or independent oversight, purpose limitation, retention, deletion, and protection against discrimination by association. These measures would strengthen criminal protection without impeding legitimate medical research, diagnostic testing, or proportionate law-enforcement activity.

## Data Availability

The original contributions presented in the study are included in the article/Supplementary Material, further inquiries can be directed to the corresponding author.
